# Identification of KRAS mutation and HER2 expression in Indonesian colorectal cancer population: a cross-sectional study

**DOI:** 10.1097/MS9.0000000000000694

**Published:** 2023-04-17

**Authors:** Reno Rudiman, Alma Wijaya, Yunia Sribudiani, Hardi Siswo Soedjana, Hesti Lina Wiraswati, Etis Primastari, Prapanca Nugraha, Kiki Lukman

**Affiliations:** Departments of a Surgery; b Pathologic Anatomy, Faculty of Medicine, Padjadjaran University/Dr. Hasan Sadikin General Hospital; c Department of Basic Medical Sciences, Faculty of Medicine, Padjadjaran University, Bandung, Indonesia

**Keywords:** colorectal cancer, epidermal growth factor receptor, human epidermal growth factor receptor-2, Kirsten rat sarcoma virus

## Abstract

In Indonesia, colorectal cancer is the third most common type. In 2008, Indonesia ranked fourth in the Association of Southeast Asian Nations (ASEAN) countries, with an incidence rate of 17.2 per 100 000 population. This figure is predicted to continue to increase from year to year. In 30% of colorectal cancer patients diagnosed after metastases, some patients will develop metastases after undergoing surgical resection of the primary tumor. The survival of metastatic colorectal cancer patients has improved significantly in the last 20 years with the introduction of target-oriented drugs, anti–epidermal growth factor receptor (EGFR), and anti–human epidermal growth factor receptor-2 (HER2). This study aims to assess the relationship between Kirsten rat sarcoma viral oncogene homolog (KRAS) mutation and HER2 expression for targeted therapy implementation.

**Patients and methods:**

This research is a cross-sectional study. The research subjects in this study were colorectal cancer patients in the digestive surgery division. There were 58 study subjects. Examination of KRAS mutations was carried out by PCR on fresh tumor tissue obtained from surgery or colonoscopy. Meanwhile, the HER2 examination used the immunohistochemistry method of paraffin blocks for anatomical pathology examination.

**Results:**

Examination of KRAS mutations showed 28/58 (43.8%) patients with colorectal cancer, while HER2 overexpression was found in 6/58 (10.3%) patients with colorectal cancer. Univariate analysis of KRAS mutations and HER2 expression showed that four subjects with KRAS mutations had excess HER2 expression (*P*=0.341).

**Conclusion:**

There is no association between KRAS mutations and HER2 overexpression in colorectal cancer patients.

## Introduction

Highlights

- In 30% of colorectal cancer (CRC) patients diagnosed after metastases, some other patients will develop metastases after undergoing surgical resection of the primary tumor.
- The survival of metastatic CRC patients has improved significantly in the last 20 years with the introduction of target-oriented drugs, anti–epidermal growth factor receptor (EGFR), and anti–human epidermal growth factor receptor-2 (HER2).
- Kirsten rat sarcoma viral oncogene homolog (KRAS) mutations are also thought to be a major negative predictor of the efficacy of anti-EGFR monoclonal antibodies.
- In CRC, HER2 overexpression has also been used as a potential therapeutic target.

The American Cancer Society states that new cases of CRC in 2021 are expected to reach 104 270 cases of colon cancer and 45 230 cases of rectal cancer, with a total of 52 980 people dying from this cancer. The incidence of CRC is 30% higher in men than in women, with the percentages for rectal cancer at 60% and colon cancer at 20%^1^. In Indonesia, CRC is the third most common type of cancer. In 2008, Indonesia ranked fourth among Association of Southeast Asian Nations (ASEAN) countries with an incidence rate of 17.2 per 100 000 population, and this figure is predicted to continue to increase from year to year^2^.

In 30% of CRC patients diagnosed after metastases, some other CRC patients will develop metastases after undergoing surgical resection of the primary tumor. The survival of metastatic colorectal cancer (mCRC) patients has improved significantly in the last 20 years with the introduction of target-oriented drugs, anti–epidermal growth factor receptor (EGFR), and antiangiogenic agents associated with chemotherapy (fluoropyrimidines, oxaliplatin, and irinotecan)^3^.

The pathogenesis of CRC is influenced by local colon conditions and individual genetics^4^,^5^. Like other types of cancer, genomic instability plays a major role in CRC, in which three distinct groups have been identified as mechanisms of pathogenesis, among them microsatellite instability, CpG island methylation phenotype, and chromosomal instability, which represent up to 80–85% of the causes of all cases of CRC^5^. The three human RAS genes (KRAS, NRAS, and HRAS) are the most frequently mutated oncogenes in human cancers, occurring in 90% of pancreatic cancers, 35% of lung cancers, and 45% of colon cancers. RAS is a component of the mitogen-activated protein kinase (MAPK) signaling pathway; this MAPK is activated by binding a ligand to a receptor tyrosine kinase such as EGFR. RAS exists in either an inactive (GDP, guanosine diphosphatase) or an active (GTP, guanine triphosphatase) state, and the transition between these two states is responsible for the signal transduction events that occur from cell surface receptors to the interior of the cell, where they are critical to the cell's growth and differentiation^5^.

KRAS is a guanosine triphosphate/guanosine diphosphate (GTP/GDP) binding protein widely expressed in most human cells. As a small GTPase (GTP-breaking enzyme), KRAS is involved in intracellular signal transduction and primarily activates MAPK-EGFR signaling. The exchange of active GTP-bound and inactive GDP-bound states is tightly controlled by GTPase activating proteins (GAP) and guanine nucleotide exchange factors^6^. Under normal physiological conditions, the signal activates KRAS-wild-type (KRAS-WT) by inducing the exchange of GDP bonds for GTP. This process is transient due to the GAP-mediated hydrolysis of GTP. However, this process becomes altered when the KRAS gene is mutated. KRAS mutations are found in 35–45% of cases of CRC. This mutation impairs KRAS’s intrinsic GTPase activity and prevents GAP from inducing GTP hydrolysis by KRAS, thereby causing KRAS protein to accumulate in its GTP-bound active form. In this way, mutant KRAS generates a constitutively active GTP-bound state, activation of proliferative signaling pathways, and tumorigenesis^7^. KRAS mutations are also thought to be a major negative predictor of the efficacy of anti-EGFR monoclonal antibodies^8^–^10^.

This relatively high percentage of RAS mutations makes RAS an important target in oncology for drug development. Therefore, mutations in KRAS, NRAS, BRAF, and PIK3CA are important predictive and prognostic markers for anti-EGFR therapy. Current guidelines have recommended that KRAS, NRAS, and BRAF mutation status be tested when considering anti-EGFR treatment. However, the rapid development of therapies for CRC patients has led to an increasing list of genes that are recommended to be examined before CRC management, such as human epidermal growth factor receptor-2 (ERBB2) and ERBB3. ERBB2, also known as HER2. HER2 is a proto-oncogene that encodes a transmembrane glycoprotein receptor with tyrosine kinase activity. HER2 is the only EGFR family member that does not bind a ligand. Still, its homodimerization or heterodimerization with other EGFR family members (HER1/EGFR, HER3, HER4) induces transphosphorylation of the intracytoplasmic tyrosine kinase domain and activation of various signal transduction pathways. Amplification of the HER2 oncogene or overexpression of its protein results in hyperactivation of mitogenic signals, even in the absence of ligand binding to other receptors, which can lead to uncontrolled cell proliferation and tumorigenesis^11^. In CRC, HER2 overexpression and amplification have also been used as potential therapeutic targets. Although several studies have reported the incidence rate of HER2 overexpression or amplification in CRC, it varies widely, ranging from 0 to 83%^12^,^13^. The biological and clinical roles of HER2 activation still need to be investigated in the pathogenesis of CRC and its association with tumor behavior, prognosis, and responses to chemotherapy, as well as with potential anti-HER2-targeted therapies^14^.

The relationship between KRAS status and HER2 also requires further investigation. One study showed that KRAS mutations and HER2 amplification were mutually exclusive^12^, while another showed no association between HER2 amplification and KRAS mutations. Thus, anti-HER2 therapy, such as trastuzumab, is thought to be a possible treatment option. for CRC with KRAS mutations. Based on the CRC management guidelines published by the National Comprehensive Cancer Network (NCCN) in 2022, anti-HER2 administration can only be done in patients with HER2 overexpression and no RAS or BRAF mutations^13^. HER2 overexpression is usually detected by immunohistochemical (IHC) analysis of the HER2 protein or fluorescence in situ hybridization analysis of the gene amplification^15^. This study aims to assess the relationship between KRAS mutation and HER2 expression to determine personalized targeted chemotherapy treatment for CRC patients, especially in the metastatic group, where the targeted therapy can be added after the chemotherapy or radiation therapy.

## Patients and methods

### Study design and setting

**Figure 1:**
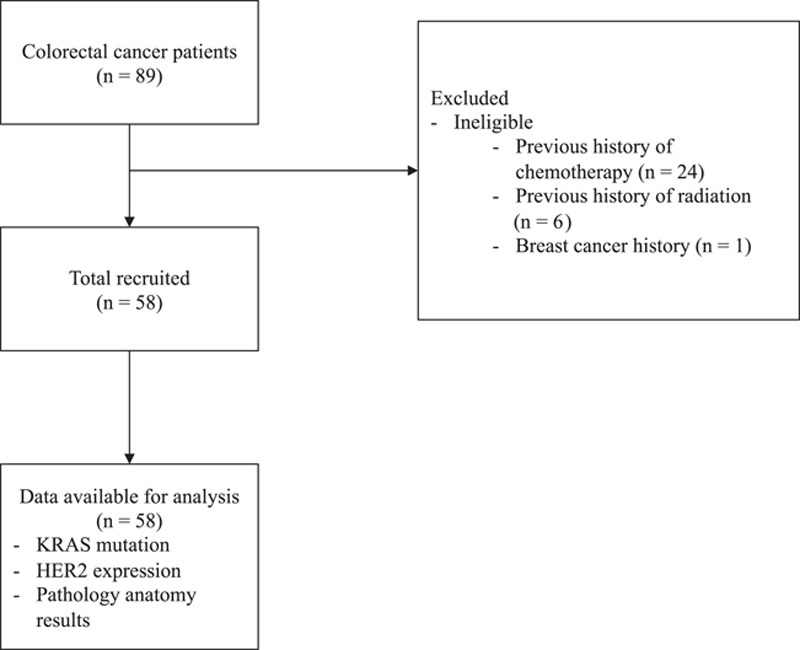
The flowchart of the study selection process.

This research is a cross-sectional study in the West Java, Indonesian population and follows the Strengthening the Reporting of Cohort Studies in Surgery (STROCSS) guidelines^16^. The research subjects in this study were CRC patients in the Digestive Surgery division of a tertiary general hospital in West Java from November 2021 to September 2022. The sample size for this study was calculated using the proportion estimation sample size, and there were 58 study subjects (Fig. 1). The inclusion criteria for this study are CRC patients with anatomical pathology confirmation who are over 18 years old and who have agreed to be the subject of this study. We excluded colorectal patients who have undergone chemotherapy and radiotherapy and have comorbidities such as breast cancer, lung cancer, and pancreatic cancer. Examination of KRAS mutations was carried out by PCR from fresh tumor tissue obtained from surgery or colonoscopy. Meanwhile, the HER2 examination used the IHC method of paraffin blocks for anatomical pathology examination. The hospital ethics committee approved the study by obtaining informed consent from the patient.

### Tumor tissue collection blood sampling and PCR analysis

Tissue samples were collected from a colonoscopy biopsy or surgical resection. Part of the tissue was immediately sent to the Pathology Anatomy Laboratory for hematoxylin–eosin staining, and the remaining tissue was stored in DNA/RNA shield solution (Zymo Research) for further genomic DNA isolation. A maximum of 10 mg of fresh colon tissues were dissected into single cells by vortexing them for 40 s using the ZR BashingBead Lysis Tube (Zymo Research). The cell suspension was centrifuged at 14 000 rpm for 30 s. Cells pellet was used for genomic DNA isolation using Quick-DNATM Miniprep (Zymo Research) according to the manufacturer’s protocol. DNA quality was measured using a NanoDropTM2000 Spectrophotometer (Thermofisher). A PCR of all exons of KRAS was performed. All exons were amplified using the touch-down PCR method with annealing temperatures ranging from 65 to 55°C. Sequencing was performed forward, and identified mutations were validated in reverse. The DNA amplification step in presequencing was performed using the Big Dye Terminator V3.3 kit (Applied Biosystem) on an ABI 3500 automated sequencer. In *silico*, an analysis of identified mutations was performed to predict the functional impact of the mutations. The analysis was performed using three online software programs: Mutation Taster 2021, PolyPhen-2, and SIFT.

### Human epidermal growth factor receptor-2 protein expression using immunohistochemical analysis

**Figure 2:**
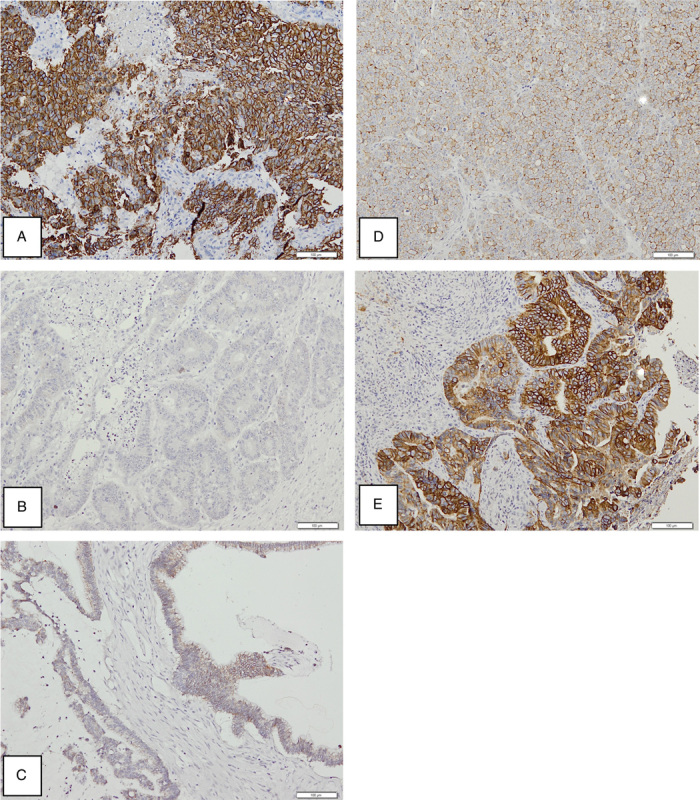
Assessment of HER2 expression with IHC, (A). Control IHC score 3+ in breast tumor tissue, (B). IHC score 0: no staining of tumor cells, (C). IHC score 1+: faint membrane staining more than 10%, granular or segmental, (D). IHC score 2+: weak to moderate membrane staining, circumferential, basolateral, or lateral membrane, more than 10% of tumor cells, (E). IHC score 3+: intense membrane staining, circumferential, basolateral, or lateral in more than 10% of tumor cells.

Tissue samples were examined histologically by hematoylin–eosin staining for the diagnosis of CRC. The subjects' block paraffin was immunohistochemically stained using the monoclonal antibody for HER2 (Brand Cell Marque, catalogue 237R-24). Two experienced pathologists (B.S.H. and H.Y.) scored independently, following the consensus recommendations for HER2 scoring for CRC, on a four-point scale (0, 1+, 2+, 3+). As far as HER2 localization is concerned, 2+ and 3+ showed predominantly membrane localization, while 1+ and 0+ showed more staining in the cytoplasm of the tumor cells, as shown in Fig. 2. Our study focused on the assessment of membranous HER2 expression. HER2 expression of +3 was identified as strongly positive or overexpression.

### Statistical analysis

The statistical analysis of the variable was performed using the SPSS 26 software (SPSS Inc.). The comparison of the variables was based on the χ^2^ test.

## Results

### Study population characteristics

As described in Table 1, 58 patients were included in the study. There were 37 women and 21 men patients. The mean age was 56.5±1.41 years, and 74.1% of patients were older than 50. Most of the patients had adenocarcinomas histologically (87.9%), and 63.8% showed well differentiation of the tumor. There were 44 (75.9%) patients with right-sided cancer and 14 (24.1%) with left-sided cancer. The right-sided cancer includes tumors in the cecum, ascending colon, and transverse colon. The left-sided tumor consists of the descending colon, sigmoid, and rectum. KRAS mutation was shown in 28 (48.3%) patients and overexpression of HER2 was shown in 6 (10.3%) patients.

**Table 1:** Characteristics of research subjects

Variables	*n* (%)
Age (years)	
Mean	56.83±1.41
Median	56.5
Age (years)	
<50	15 (25.9)
>50	43 (74.1)
Sex	
Man	21 (36.2)
Woman	37 (63.8)
Histologic	
Adenocarcinoma	51 (87.9)
Mucinous adenocarcinoma	5 (8.6)
Signet ring cell	1 (1.7)
Neuroendocrine	1 (1.7)
Grade	
Well-differentiated	37 (63.8)
Moderately differentiated	7 (12.1)
Poorly differentiated	7 (12.1)
Specific	7 (12.1)
Tumor location	
Right side	14 (24.1)
Cecum	4 (6.8)
Ascending colon	4 (6.8)
Transverse colon	6 (10.3)
Left side	44 (75.9)
Descending colon	2 (3.4)
Sigmoid	5 (8.6)
Rectum	37 (63.7)
Stage	
I	1 (1.7)
II	12 (20.7)
III	17 (29.3)
IV	28 (48.3)
Metastases	
Liver	16 (27.6)
Lung	3 (5.2)
Bone	1 (1.7)
Omentum	1 (1.7)
Perforation	1 (1.7)
Uterine	1 (1.7)
Liver and bone	2 (3.4)
Liver and lung	1 (1.7)
KRAS	
Mutation	28 (48.3)
Wild-type	30 (51.7)
HER2	
0	23 (39.7)
+	12 (20.7)
++	19 (29.3)
+++	6 (10.3)
HER2	
Normal expression	52 (89.7)
Overexpression (+++)	6 (10.3)

HER2, human epidermal growth factor receptor-2; KRAS, Kirsten rat sarcoma virus.

### Univariate analysis of variables and Kirsten rat sarcoma virus mutation

In univariate analysis among seven variables related to the KRAS mutation, none were statistically significant (*P*<0.05), as shown in Table 2.

**Table 2:** Univariate analysis for subject characteristics with Kirsten rat sarcoma virus mutations

	*n* (%)		
	Mutation	Wild-type	*P* (χ^2^)
Age (years)			
Early onset (<50)	9 (15.5)	6 (10.3)	0.29
Late onset (>50)	19 (32.8)	24 (41.4)	
Sex			
Man	10 (17.2)	11 (19)	0.94
Woman	18 (31.0)	19 (32.8)	
Histologic			
Adenocarcinoma	26 (44.8)	25 (43.1)	
Mucinous adenocarcinoma	1 (1.7)	4 (6.9)	0.28
Signet ring cell	1 (1.7)	0	
Neuroendocrine	0	1 (1.7)	
Grade			
Well-differentiated	18 (31.0)	19 (32.8)	
Moderately differentiated	5 (8.6)	2 (3.4)	0.44
Poorly differentiated	3 (5.2)	4 (6.9)	
Specific	2 (3.4)	5 (8.6)	
Tumor location			
Right side	6 (10.3)	8 (13.8)	0.09
Left side	22 (37.9)	22 (37.9)	
Stage			
I	0	1 (1.7)	
II	4 (5.8)	8 (13.8)	0.48
III	9 (15.5)	8 (13.8)	
IV	15 (26.3)	13 (22.4)	
HER2			
Normal expression	24 (41.4)	28 (48.3)	
Overexpression (+++)	4 (6.9)	2 (3.4)	0.34

HER2, human epidermal growth factor receptor-2.

### Univariate analysis of variables and human epidermal growth factor receptor-2 expression

In univariate analysis of the variables to the HER2 expression, one was statistically significant (*P*<0.05), as shown in Table 3. There were five (8.6%) subjects in stage II with overexpression of HER2 with *P*-value equal to 0.001.

**Table 3:** Univariate analysis for subject characteristics with human epidermal growth factor receptor-2 expression

	*n* (%)		
	Overexpression	Normal	*P* (χ^2^)
Age (years)			
Early onset (<50)	2 (3.4)	13 (22.4)	0.65
Late onset (>50)	4 (6.9)	39 (67.2)	
Sex			
Man	1 (1.7)	20 (34.5)	0.29
Woman	5 (8.6)	32 (55.2)	
Histologic			
Adenocarcinoma	5 (8.6)	46 (79.3)	
Mucinous adenocarcinoma	1 (1.7)	4 (6.9)	0.86
Signet ring cell	0	1 (1.7)	
Neuroendocrine	0	1 (1.7)	
Grade			
Well-differentiated	4 (6.9)	33 (56.9)	
Moderately differentiated	0	7 (12.1)	0.78
Poorly differentiated	1 (1.7)	6 (10.3)	
Specific	1 (1.7)	6 (10.3)	
Tumor location			
Right side	0	14 (24.1)	0.14
Left side	6 (10.3)	38 (65.5)	
Stage			
I	0	1 (1.7)	
II	5 (8.6)	7 (12.1)	0.001
III	0	17 (29.3)	
IV	1 (1.7)	27 (46.6)	
KRAS			
Wild-type	2 (3.4)	28 (48.3)	0.34
Mutation	4 (6.9)	24 (41.4)	

KRAS, Kirsten rat sarcoma virus.

## Discussion

This is a single-center retrospective cross-sectional study describing and analyzing KRAS mutation and HER expression in CRC patients. Table 1 shows that the age of the research subjects was an average of 56.83±1.41 years and a median of 56.5 years. This shows that patients who come to the hospital with a diagnosis of CRC are mostly over 50 years old. Based on epidemiological studies, the results of this study are consistent with the profile of patients with CRC, most of whom are over 50 years old. Data showing that the risk of CRC increases in the fifth decade of life has been widely cited. In some studies, the risk increases by up to 1% for each additional 10 years starting at age 50^17^,^18^. Based on sex, there were 37 women (63.8%) and 21 men (36.2%). There were more women subjects than men. In another study, there were no differences in colorectal prevalence between sexes, with a prevalence rate of 1:23 (4.3%) in men and 1:25 (4.0%) in women. Based on the results of anatomical pathology (histologic) examination, as many as 51 subjects (87.9%) showed adenocarcinoma, followed by mucin adenocarcinoma in five subjects (8.6%), signet ring cell carcinoma and neuroendocrine carcinoma, each in one subject (1.7%). Meanwhile, based on grade, 37 subjects (63.8%) showed good differentiation, while moderate and poor differentiation, respectively, seven subjects (12.1%). Based on literature studies, the type of adenocarcinoma in CRC is found in up to 90% of the total cases. The differentiation of the adenocarcinoma itself can be in the form of comedo, medullary, micropapillary, mucinous, or signet ring cells^17^,^18^. In the adenocarcinoma type, most of the literature studies show moderate grade or differentiation, which is as much as 70%, followed by well and poor differentiation of around 10–20%^19^.

Based on the tumor's location, there were 44 (74.9%) cancers in the descending colon, sigmoid, and rectum and 14 (24.1%) cancers in the cecum, ascending colon, and transverse colon. Based on the existing literature, about 70% of total colon cancer occurs in the descending colon (10% descending colon, 10% sigmoid, and 50% rectum), and then about 29.5% occur in the ascending and transverse colon^17^,^18^. Most of the study subjects, namely 28 subjects (48.3%), suffered from CRC stage IV, with the most metastases in the liver, namely 16/28 (57.1%), followed by 17 subjects (29.3%) suffering from stage III, 12 subjects (20.7%) in stage II, and one subject (1.7%) in stage I. This shows that patients with CRC in the Indonesian population seek medication when there are already significant clinical symptoms such as constipation, blood in the stool, and abdominal pain^20^.

KRAS mutations in this study showed 28 subjects (48.3%) with positive mutation results. In this study, the proportion of KRAS mutations was higher than in several other studies. KRAS mutation is one of the oncogenes that have mutations in 35–45% of CRCs^21^–^24^. In a study in Indonesia, Abdullah *et al*.^25^ showed KRAS mutations in 16.3% of patients with CRC, while Levi’s *et al*.’s^26^ showed a KRAS mutation of 34.9%. The study by Ross *et al*.^27^ showed that 51.6% of the subjects of 7599 colon cancer patients showed the KRAS mutation, and 53% of the subjects of 1288 rectal cancer patients showed the KRAS mutation. Univariate analysis of KRAS mutations did not show a significant association between KRAS mutations and age, sex, type of anatomic pathology, grade, stage, or location of the tumor. In a study by Zannato *et al*.^28^, in 2020, the KRAS mutation was found in 43.4% of CRC patients with no significant relationship with age, sex, stage, or metastasis. In a study by Dai *et al*.^29^, in 2020, from 8983 CRC patients, KRAS mutations were found in 40.2% and showed significant association with stage III and IV, left-sided colon, and rectum (*P*<0.001). In a study by Alghamdi *et al*.^30^, in 2022, the KRAS mutation shown in 50% of CRC patients has a significant association with the right-sided tumor and with peritoneal metastases, but other clinicopathological characteristics show no significant association.

HER2 expression in this study showed six subjects (10.3%) with overexpression results. In this study, the proportion of overexpression of HER2 appeared to be higher than in several other studies. In the study of Seo and colleagues, in 2014, there were two HER2 assessment cohorts: the first cohort involved 365 patients with CRC, and HER2 overexpression was found in eight subjects (2.2%). The second cohort involved 174 patients with stage IV CRC, and HER2 overexpression was present in five subjects (2.9%)^31^. Valtorta *et al*.^15^ in 2015 conducted a study on 304 patients with CRC. 14 subjects (4.6%) showed HER2 overexpression. The study by Ross *et al*.^27^, in 2018 showed that 140 subjects (1.6%) of 8887 colorectal patients showed excess HER2 expression from metastatic CRC. Razzaq *et al*.^32^, in 2021 assessed HER2 expression in patients with CRC; out of 17 patients with CRC, there were four subjects (23.52%) with excess HER2 expression. Univariate analysis of HER2 expression did not show a significant relationship between HER2 overexpression and age, sex, type of anatomic pathology, grade, or tumor location. A significant association was only seen at the tumor stage; most of the HER2 overexpression was at stage II (*P*=0.001). In the study by Kamal and Jalal^33^, in 2019, HER2 overexpression was found in 53.4% of CRC patients and showed a significant association with the grade of the tumor but no association with age, sex, or tumor site. In a study by Işik and Barut^34^, in 2020 on 123 CRC patients, HER2 overexpression was found in 13% of patients, and HER2 overexpression showed a significant association with distant metastasis (*P*<0.05) and showed no relationship with age, sex, tumor site, or grade of the tumor. In a study by Kaur *et al*.^35^, in 2020, HER2 overexpression was found in 32% of CRC patients with no association regarding age, sex, or tumor size.

Univariate analysis of KRAS mutations with HER2 overexpression showed a *P*-value equal to 0.34. This indicated no significant relationship between KRAS mutations and HER2 overexpression. A cross-tabulation of HER2 expression and KRAS mutations showed that out of 28 subjects showing KRAS mutations, only four subjects (6.9%) had HER2 overexpression. A study by Bar and colleagues showed KRAS mutation in 46 CRC patients in 28.2–34.7% and HER2 overexpression in 80.3% of patients. The patient with KRAS mutation and HER2 overexpression was in the subgroup of CRC with poorly differentiated grades and showed no significant association with other clinicopathological characteristics^36^. A study conducted by Ross and colleagues. In 2018, 51.6% of subjects among 7599 colon cancer patients showed KRAS mutations, and overexpression of HER2 was shown in 62.5% of mutated KRAS patients (*P*=0.02). There were 53% of the subjects of 1288 rectal cancer patients who showed KRAS mutation, and overexpression of HER2 was shown in 53.6% of those with mutated KRAS, with a *P*-value showing no significant association. The relationship between KRAS mutations and HER2 overexpression showed no significant results^27^. The study by Sawada *et al*.^37^, in 2018, based on data from 2005 to 2015, showed that only 11 subjects (2.9%) of 370 mCRC had overexpression of HER2 with RAS and BRAF wild-type, and HER2 may be a predictive factor for anti-EGFR therapy. In a study by Martianov and colleagues with a large sample size of 8.355, KRAS mutation was found in 49.5%, while HER overexpression was only shown in a small number of 99/8008 (1.2%). Patients with KRAS mutations and HER2 overexpression were only shown in 0.3% of patients, and the relationship between KRAS mutations and HER2 overexpression showed no significant results^38^.

The limitations of the study are the relatively small sample size and the fact that the study did not collect information regarding the treatment outcome because the recruited patients had not finished undergoing chemotherapy or radiation during the study period. KRAS examination in CRC is very important to determine the appropriate therapy for the patient and the effectiveness of the therapy to be carried out. An example is the use of anti-EGFR in CRC, which is said to be effective in these patients but cannot be done if there is a mutation in KRAS. This proves that mutations in KRAS signify a poor prognosis in CRC patients^5^,^7^,^39^. The benefit of this study is to prove that KRAS mutational status and HER2 expression status in CRC patients can help to deciding targeted therapy, especially for mCRC. In conclusion, KRAS and HER2 expression are independent factors in tumorigenesis and have no association. The presence of KRAS mutation and HER2 overexpression in CRCs suggests a possible role for the use of specific treatments such as anti-EGFR and anti-HER2. Implementing targeted therapy in mCRC requires evaluating all the components involved in the MAPK pathway, like the RAS family and BRAF mutational status. In the near future, the status of HER2 in mCRC may change the algorithm of mCRC treatment toward an increasingly personalized therapeutic approach.

## Ethical approval

The Dr. Hasan Sadikin Hospital Ethics Committee approved the study with No. Ethical Approval LB.02.01/X.6.5/326/2022. The Dr. Hasan Sadikin Hospital Ethics Committee approved the study. Every research participant signed an informed consent before participating in the study.

## Consent

The Dr. Hasan Sadikin Hospital Ethics Committee approved the study with a waiver of informed consent. Every research participant signed an informed consent before participating in the study. A copy of the written consent is available for review by the Editor-in-Chief of this journal on request.

## Sources of funding

The preparation of this study was supported by internal funding from a Research Grant from Riset Kompetensi Dosen Unpad (RKDU) Faculty of Medicine, Universitas Padjadjaran with registered number 2203/UN6.3.1/PT.00/2022.

## Author contribution

All authors participated in the conception and design of the study, drafting, and finalizing the manuscript. A.W., K.L., and P.N. participated in collecting the patient's information. Y.S. and H.L.W. participated in analyzing the KRAS mutation. E.P. participated in the histologic examination of the colorectal diagnosis and IHC examination of HER2. R.R. and H.S.S. analyzed the data and interpretation.

## Conflicts of interest disclosure

The authors declare that they have no financial conflict of interest with regard to the content of this report.

## Research registration unique identifying number (UIN)

Not applicable.

## Guarantor

Reno Rudiman.

## Data availability statement

All data and tables used to support the findings of this study are included within the article and available upon request to corresponding author.

## Provenance and peer review

Not commissioned, externally peer reviewed.
